# Bilaterality in papillary thyroid carcinoma: long-term outcomes and predictive factors

**DOI:** 10.1186/s13044-026-00285-8

**Published:** 2026-01-12

**Authors:** Jingwen Zhang, Mengjia Fei, Wenhan Wang, Yijie Dong, Jianqiao Zhou

**Affiliations:** 1 Department of Ultrasound, Ruijin Hospital, School of Medicine, Shanghai Jiao Tong University Shanghai, Shanghai, 200025 China; 2 Department of Head and Neck Surgery, Renji Hospital, School of Medicine, Shanghai Jiao Tong University Shanghai, Shanghai, 200001 China

**Keywords:** Papillary thyroid carcinoma, Bilaterality, Prognosis, Recurrence, Total thyroidectomy

## Abstract

**Purpose:**

Bilaterality in papillary thyroid carcinoma (PTC) is a critical factor influencing surgical approach selection. This study aimed to investigate the long-term outcomes and predictive factors associated with bilaterality in PTC.

**Methods:**

This retrospective study reviewed 2,816 consecutive PTC patients who underwent total thyroidectomy between January 2013 and December 2015. Risk factors for bilaterality were analyzed using multivariate logistic regression. Among these, 1282 patients with ≥ 24-months follow-up were included in further recurrence/persistence analysis. The primary outcome measured was recurrence-free survival (RFS).

**Results:**

Bilaterality was significantly associated with shorter RFS compared to unilateral PTC (*p* < 0.01), with a mean follow-up of 74.3 ± 33.3 months (median: 71 months; range: 24–140 months). Additionally, multifocality (OR = 4.58, *p* < 0.01), advanced N stage (N1a: OR = 1.36, *p* = 0.002; N1b: OR = 1.61, *p* < 0.01), obesity (OR = 1.74, *p* < 0.01), and a family history of thyroid cancer (OR = 2.16, *p* < 0.01) were independent predictors of bilaterality.

**Conclusion:**

A comprehensive preoperative examination should be conducted to detect bilaterality, particularly in PTC patients with identified risk factors. Additionally, intensive postoperative follow-up is recommended for patients with bilaterality to monitor for recurrence/persistence.

## Introduction

The incidence of papillary thyroid carcinoma (PTC) has increased rapidly due to diagnostic advancements [1]. Despite its favorable prognosis, PTC recurrence rates range from 4.3% to 35%, potentially leading to adverse outcomes [2–4]. PTC can present with two or more distinct foci within the thyroid lobes, and bilaterality, defined as the presence of two or more distinct malignant foci within both thyroid lobes, is reported in 13% to 71% [5–7]. Emerging evidence suggests that bilaterality is associated with more aggressive disease in PTC, and it is also an independent predictor of recurrence [2, 7–9].

The 2015 American Thyroid Association (ATA) guidelines suggest lobectomy for PTCs < 1 cm and either lobectomy or total thyroidectomy for tumors 1–4 cm without extrathyroidal extension (ETE) or lymph node metastasis (LNM) [10]. However, studies indicate approximately 50% of patients with PTC undergoing unilateral resection may harbor residual cancer in the contralateral lobe [11, 12]. These findings emphasize the critical importance of accurate preoperative assessment of bilaterality. Despite prior studies to identify clinicopathological predictors of bilaterality, findings have been inconsistent [7, 13, 14]. Furthermore, patient-specific factors, such as obesity and family history, may also influence bilaterality risk. Few large-scale studies have simultaneously combined these diverse variables into a comprehensive multivariate analysis.

Therefore, this large-scale retrospective study aims to: (1) evaluate the long-term prognostic impact of bilaterality in PTC patients; and (2) identify reliable demographics and clinicopathological predictors of bilaterality to inform individualized surgical planning.

## Materials and methods

### Patient selection

We retrospectively analyzed consecutive patients with pathologically confirmed PTC who underwent total/near-total thyroidectomy at our institution between January 2013 and December 2015. The decision to perform a total/near-total thyroidectomy was based on one or more of the following preoperative findings: (1) Indications for total thyroidectomy in accordance with 2009 ATA guidelines; (2) US evidence of bilateral nodules (suspicious malignant or large benign nodules in the contralateral lobe). (3) Fine-needle aspiration (FNA) cytology suspicious for or diagnostic of malignancy in both thyroid lobes; (4) family history or genetic predisposition warranting more extensive surgery; (5) surgeon’s decision based on preoperative/intraoperative conditions or patient preference after counseling.

Exclusion criteria included: (1) age < 18 years; (2) previous thyroid surgery; (3) mixed histology (coexisting follicular, medullary, or anaplastic carcinoma); (4) distant metastasis at diagnosis; (5) PTC > 4 cm (as guidelines uniformly recommend total thyroidectomy regardless of bilaterality status). A total of 2816 PTC cases were included in this study. Among these, 1,282 patients with negative initial postoperative US findings and ≥ 24-month follow-up were eligible for recurrence/persistence predictor evaluation. Recurrence/persistence was defined as the detection of new locoregional lesions or distant metastases during the postoperative period using ultrasound, CT, or other imaging modalities.

The retrospective study was performed in accordance with the Declaration of Helsinki and approved by the Ethics Committee of Shanghai Jiao Tong University Affiliated Ruijin Hospital, and informed consent was not required due to retrospective design.

### Clinical-pathological data

Patient demographics and tumor characteristics were collected: age, gender, tumor size, multifocality, bilaterality, obesity (defined as a body mass index (BMI) ≥ 30 kg/m²), family history of thyroid cancer in first-degree relatives (parents, offspring, siblings), Hashimoto’s thyroiditis, and pathological tumor T and N stages. Multifocality was defined as the presence of more than one focus within a single thyroid lobe.

Tumor staging was performed according to the 8th edition of American Joint Committee on Cancer (AJCC) staging system [15]. For all patients, we recorded the duration of follow-up. In cases with tumor recurrence/persistence (TRP), we documented the interval between initial surgery and first recurrence detection.

### Follow-up protocol and study endpoint

Postoperative follow-up protocol was conducted as follows: patients underwent physical examinations and laboratory tests, including serum thyroid function, thyroglobulin, and anti-thyroglobulin antibody levels. TSH suppression therapy was administered in accordance with the American Thyroid Association (ATA) management guidelines for patients with differentiated thyroid cancer [10]. Neck ultrasonography was performed at 3, 6, and 12 months postoperatively and annually thereafter. Additional imaging including computed tomography (CT), positron emission tomography/CT (PET/CT), or whole-body RAI scan was performed when clinically indicated. The primary endpoint was recurrence-free survival (RFS), defined as the time from initial surgery to first histologically or radiologically confirmed recurrence. In cases without recurrence/persistence, the last follow-up date served as the endpoint.

### Statistical analysis

All statistical analyses were conducted using SPSS 24.0 (IBM Corp., Armonk, NY). Continuous variables were presented as mean ± standard deviation, and categorical variables as frequencies (percentages). Group Comparisons were performed using χ² tests or Fisher’s exact tests for categorical variables and independent samples t-test or Mann-Whitney U test for continuous variables. Univariate and multivariate logistic regression identified predictors of bilaterality and recurrence/persistence. Survival analysis used the Kaplan-Meier method with log-rank test. *P* < 0.05 was considered statistically significant.

## Results

### Patient characteristics

Clinicopathological characteristics are summarized in Table 1. The total cohort had a mean age of 44.8 ± 12.4 years (range: 18–82) and was predominantly female (75.4%; 2,124/2,816).Mean tumor diameter was 11.5 ± 7.0 mm (range: 1.9–40.0). In the followed-up cohort (*n* = 1,282), the TRP-positive group was significantly younger (41.0 ± 13.6 years vs. 45.1 ± 12.2 years, *p* < 0.01), and the two groups showed a significant difference in sex distribution (*p* < 0.01). On pathological examination, the TRP-positive group had significantly larger tumors (15.6 ± 8.8 mm vs. 11.5 ± 7.1 mm, *p* < 0.001). Bilaterality (47.8% vs. 29.7%, *p* < 0.01) and multifocality (35.4% vs. 20.4%, *p* < 0.01) were more frequently observed in the TRP-positive group. Moreover, patients with TRP-positive had a higher proportion of more advanced T stage (*p* < 0.001) and N stage (*p* < 0.001). However, the two groups did not significantly differ in the presence of concurrent Hashimoto’s thyroiditis (*p* = 0.13), family history of thyroid cancer (*p* = 1.00), or obesity (*p* = 0.35).

**Table 1:** Clinicopathological characteristics of included PTC patients

Clinicopathologic characteristics	No. of Patients (%)			
	Total *n* = 2816	Follow up ≥ 24 months (*n* = 1282)		P for Recurrence/ Persistence
		TRP (+) *n* = 113	TRP (-) *n* = 1169	
Gender				< 0.01
Male	692 (24.6%)	42 (37.2%)	276 (23.6%)	
Female	2124 (75.4%)	71 (62.8%)	893 (73.4%)	
Age (years)	44.8 ± 12.4 (18–82)	41.0 ± 13.6(18–82)	45.1 ± 12.2 (19–78)	< 0.01
Tumor Size (mm)				
Mean ± SD	11.5 ± 7.0 (1.9–40.0)	15.6 ± 8.8 (3.6–40.0)	11.5 ± 7.1 (2.2–40.0)	< 0.01
unilateral	1941 (68.9%)	59 (52,2%)	822 (70.3%)	
Bilaterality	875 (31.1%)	54 (47.8%)	347 (29.7%)	< 0.01
Multifocality	612 (21.7%)	40 (35.4%)	238 (20.4%)	< 0.01
Hashimoto’s thyroiditis	315 (11.2%)	10 (8.6%)	164 (14.0%)	0.13
T stage				< 0.01
T1	2303 (81.8%)	76 (67.3%)	981 (83.9%)	
T2	208 (7.4%)	13 (11.5%)	109 (9.3%)	
T3	169 (6.0%)	9 (7.9%)	34 (2.9%)	
T4	136 (4.8%)	15 (13.3%)	45 (3.9%)	
N stage				< 0.01
N0	1486 (52.8%)	22 (19.5%)	602 (51.5%)	
N1a	973 (34.5%)	40 (35.4%)	413 (35.3%)	
N1b	357 (12.7%)	51 (45.1%)	154 (13.2%)	
Family thyroid cancer history	66 (2.3%)	3 (2.7%)	35 (3.0%)	1.00
BMI ≥ 30Kg/m^2^	128 (4.5%)	7 (6.2%)	50 (4.3%)	0.35

TRP (+): Tumor recurrence/persistence positive

TRP (-): Tumor recurrence/persistence negative

### Recurrence/persistence patterns and predictive factors in PTC

**Fig. 1:**
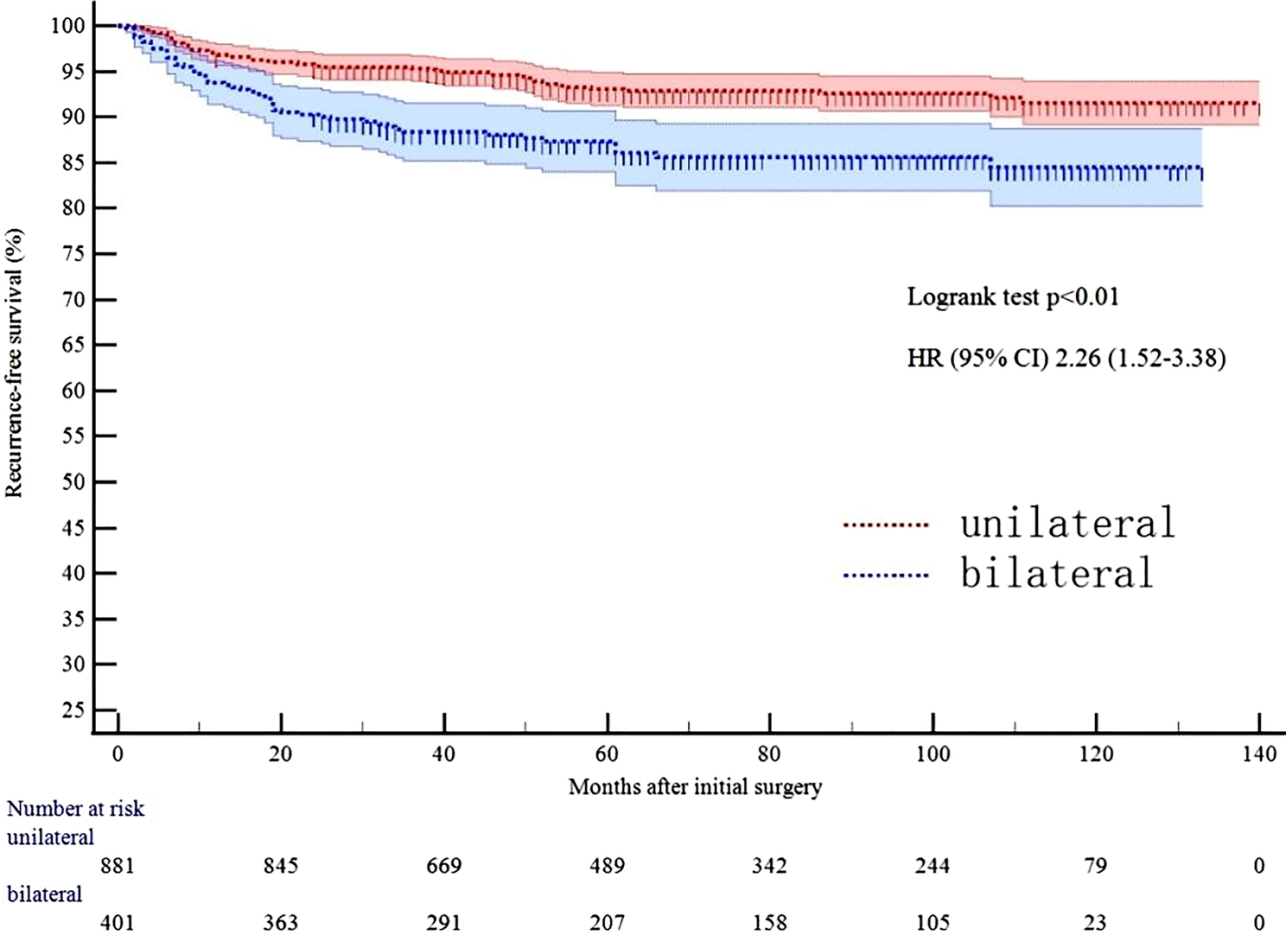
Recurrence-free survival in thyroid papillary carcinoma. Kaplan-Meier survival curve showing shorter RFS in bilaterality compared to unilateral PTC (*p* < 0.01)

Among 1,282 patients with ≥ 24-month follow-up, median follow-up was 71 months (mean 74.3 ± 33.3 months; range 24–140 months). In this cohort, 113 patients (8.8%) developed recurrence/persistence: 109 (96.4%) had regional recurrence (7 thyroid bed, 100 lymph nodes), and 4 had distant metastasis (3 pulmonary, 1 osseous). All regional recurrences/persistence were pathologically/cytologically confirmed. Distant metastases were diagnosed based on elevated thyroglobulin levels coupled with positive findings on iodine-131 scans. Multivariate regression analysis identified larger tumor size (*p* = 0.04), multifocality (*p* = 0.04), lymph node metastasis (N1a: *p* < 0.01; N1b: *p* < 0.01), and bilaterality (*p* < 0.01) as independent predictors of TRP positive (Table 2). Furthermore, bilaterality was associated with significantly shorter RFS in Kaplan-Meier analysis (*p* < 0.01; Fig. 1).

**Table 2:** Univariate and multivariate analysis for predictors of recurrence/persistence (*n* = 1282)

Variables	Univariate				Multivariate	
	OR (95%CI)	*P*			OR (95%CI)	*P*
Gender						
Male	Ref.				/	/
Female	0.52 (0.35–0.78)	< 0.01			/	/
Age	0.97 (0.96–0.99)	< 0.01			/	/
Family Thyroid Cancer History						
Absent	Ref.				/	/
Present	0.88 (0.27–2.92)	0.84			/	/
Tumor Size (mm)	1.06 (1.04–1.08)	< 0.01			1.03 (1.01–1.07)	0.04
BMI (Kg/m^2^)						
≤ 30	Ref.				/	/
> 30	1.48 (0.65–3.34)	0.35			/	/
T Stage						
T1	Ref.				Ref.	
T2	1.54 (0.83–2.86)	0.17			0.70 (0.32–1.51)	0.36
T3	3.42 (1.58–7.39)	< 0.01			2.14 (0.89–5.16)	0.09
T4	4.30 (2.29–8.07)	< 0.01			1.85 (0.89–3.85)	0.10
N Stage						
N0	Ref.				Ref.	
N1a	2.65 (1.55–4.53)	< 0.01			2.13 (1.23–3.72)	< 0.01
N1b	9.06 (5.33–15.40)	< 0.01			5.82 (3.25–10.43)	< 0.01
Hashimoto’s thyroiditis						
Absent	Ref.				/	/
Present	0.59 (0.30–1.16)	0.13			/	/
Multifocality					/	/
Absent	Ref.				Ref.	
Present	2.14 (1.42–3.23)	< 0.01			1.62 (1.01–2.58)	0.04
Bilaterality						
Absent	Ref.				Ref.	
Present	2.17 (1.47–3.20)	< 0.01			1.80 (1.16–2.79)	< 0.01

OR, odds ratio; CI, confidence interval

### Comparison of bilateral and unilateral PTC in 2816 patients

Given the prognostic value of bilaterality, we analyzed its predictors in the overall cohort (*n* = 2,816). Multivariate logistic regression revealed multifocality (OR = 4.85, *p* < 0.01), obesity (OR = 1.74, *p* < 0.01), family history of thyroid cancer (OR = 2.16, *p* < 0.01), and N1 stage (N1a: OR = 1.36, *p* < 0.01; N1b: OR = 1.61, *p* < 0.01) as independent predictors of bilaterality (Table 3).

**Table 3:** Comparison of clinicopathological characteristics between unilateral and bilaterality groups

Characteristics	Unilateral (*n* = 1941)	Bilaterality (*n* = 875)	*p*	Multivariate analysis		
				OR (95% CI)	*p*	
Gender			0.71			
Male	473 (24.4%)	219 (25%)		/		
Female	1468(75.6%)	656 (75%)		/		/
Age (years)	45.8 ± 12.4	45.8 ± 11.6	0.92	/		/
Tumor size (mm)	11.5 ± 7.1 (1.9 ~ 40.0)	11.3 ± 6.6 (2.2 ~ 40.0)	0.39	/		/
Multifocality			< 0.01			
Absent	1696 (87.4%)	508 (58.1%)		Ref.		
Present	245 (12.6%)	367 (41.9%)		4.85 (4.00–5.88)		< 0.01
Hashimoto’s thyroiditis			0.89			
Absent	1725(88.9%)	776 (88.7%)		/		/
Present	216(11.1%)	99 (11.3%)		/		/
T stage			0.03			
T1	1589 (81.9%)	714 (81.6%)		Ref.		
T2	153 (7.9%)	55 (6.3%)		0.70 (0.32–1.51)		0.36
T3	119 (6.1%)	50 (5.7%)		0.99 (0.67–1.44)		0.94
T4	80 (4.1%)	56 (6.4%)		1.34 (0.90–2.01)		0.15
N stage			< 0.01			
N0	1080 (55.6%)	407 (46.5%)		Ref.		
N1a	639 (33.0%)	333 (38.1%)		1.36 (1.12–1.65)		< 0.01
N1b	222 (11.4%)	135 (15.4%)		1.61 (1.21–2.13)		< 0.01
Family thyroid cancer history			0.01			
Absent	1905 (98.1%)	845 (96.6%)		Ref.		
Present	36 (1.9%)	30 (3.4%)		2.16 (1.28–3.63)		< 0.01
BMI(Kg/m^2^)			< 0.01			
< 30	1868 (96.2%)	820 (93.7%)		Ref.		
≥ 30	73 (3.8%)	55 (6.3%)		1.74 (1.17–2.56)		< 0.01

OR, odds ratio; CI, confidence interval

## Discussion

In this large-scale retrospective study with extended follow-up, we identified bilaterality as an independent risk factor for TRP (OR = 1.80, 95% CI: 1.16–2.79) and shorter RFS in PTC. Our findings reinforce the importance of incorporating bilaterality into preoperative risk stratification and surgical decision-making.

In this study, the incidence of bilaterality was 31.1%, which is consistent with prior reports [6–8, 16, 17]. The prognostic impact of bilaterality remains controversial. Some studies identified bilaterality as a significant predictor of recurrence in low-risk PTC patients [18–20], while Kim et al. [21] found no link between bilaterality and recurrence (OR = 0.98, 95% CI: 0.64–1.48). Similarly, Yan et al. [22] observed bilaterality correlated with extrathyroidal extension (ETE), and advanced TNM stage but not with prognosis. These discrepancies likely stem from variations in study cohorts (e.g., differing tumor size distributions or risk profiles) and, critically, in the extent of initial surgery (lobectomy vs. total thyroidectomy). Importantly, our study included only patients who underwent total or near-total thyroidectomy to avoid missing undetected contralateral malignancy by preoperative imaging.

We further identified key predictors of bilaterality. In addition to confirming previously reported risk factors such as multifocality (OR = 4.85, *p* < 0.01) and advanced N stage (N1a: OR = 1.36, *p* < 0.01; N1b: OR = 1.61, *p* < 0.01), this study provides novel insights by identifying obesity (OR = 1.74, *p* < 0.01) and family history of thyroid cancer (OR = 2.16, *p* < 0.01) as predictors—both of which have been underexplored in prior work.

The mechanism between obesity and bilaterality is not yet fully clearly understood, but several biologically plausible pathways have been proposed. One potential pathway involves obesity-related hyperinsulinemia. This condition may promote thyroid cell proliferation via insulin-like growth factor signaling [23]. Additionally, adipose tissue-derived cytokines may create a pro-inflammatory microenvironment, which may facilitate tumor development [24]. Some studies associate elevated BMI with bilaterality [24, 25], while others do not consistently identify obesity as a predictor [26]. Thus, further investigation of the association mechanism is needed.

The role of family history was particularly pronounced, with a significantly higher prevalence of bilaterality (45.4% vs. 30.7%, *p* < 0.05). Compared to the general population, first-degree relatives of thyroid cancer patients carry a higher risk of thyroid carcinoma [27, 28]. Additionally, familial non-medullary thyroid cancer (FNMTC) shows a significantly higher rate of bilaterality compared to sporadic cases [29]. These findings point clearly to genetic factors playing an important role. Possible explanations include intraglandular dissemination or inherited pathogenic variants [30–33]. Other possible mechanisms explaining the familial association include an unidentified environmental factor [34].

Clinically, our findings contribute to surgical planning under the ATA framework. For patients with ≤ 4 cm tumors and risk factors for bilaterality, initial total thyroidectomy may be considered to avoid subsequent completion surgery. Conversely, in low-risk tumors without such factors, lobectomy remains adequate.

This study benefits from a large sample size, comprehensive clinicopathological data, and long-term follow-up. However, several limitations should be acknowledged. First, the retrospective, single-center nature of this study may have introduced selection bias and prevented causal inference. Second, molecular markers such as BRAF V600E and TERT—linked to aggressive disease, bilaterality, and poor prognosis [14, 35]—were not routinely tested during the early phase of this long-term study. Third, RAI treatment data were incomplete. Since RAI reduces recurrence risk, this gap may have biased our risk estimates—particularly if high-risk patients received RAI, which could weaken the observed associations. Finally, due to the retrospective nature of this study, distinguishing between “persistence” and “recurrence” strictly according to ATA guidelines was challenging based solely on current imaging or biochemical tests.

In conclusion, bilaterality is an independent predictor of PTC recurrence/persistence. Multifocality, advanced nodal stage, family history of thyroid cancer, and obesity were found to be predictors of bilaterality. Patients with one or more of these factors may benefit from total thyroidectomy as the initial surgical approach. For high-risk patients with bilaterality who have undergone lobectomy, we advocate for an intensive surveillance strategy, to enable early detection and management of recurrence in the contralateral lobe.

## Data Availability

The datasets used and analyzed during the current study are available from the corresponding author on reasonable request.
